# “La ruptura en el cuidado”: Experiencias de mujeres madres con abuso de alcohol y su atención en México

**DOI:** 10.18294/sc.2023.4334

**Published:** 2023-05-30

**Authors:** Nancy Araceli Méndez Romero

**Affiliations:** 1 Doctora en Ciencias en Salud Colectiva. Catedrática, Universidad Tecnológica de México, Campus Los Reyes, Estado de México, México. nmr2500@yahoo.com.mx Universidad Tecnológica de México Universidad Tecnológica de México Estado de México México nmr2500@yahoo.com.mx

**Keywords:** Mujeres, Abuso de Alcohol, Maternidad, Cuidados, Alcohólicos Anónimos, México, Women, Alcohol Abuse, Mothers, Caring, Alcoholics Anonymous, Mexico

## Abstract

Este artículo se propone analizar las experiencias sobre la maternidad y los cuidados de mujeres madres que asisten a grupos de apoyo mutuo en la Ciudad de México y el Estado de México para autoatender los daños asociados al alcohol. A partir del marco conceptual de la salud colectiva y la perspectiva de género, se concibe que la condición de género y socioeconómica inciden en la determinación social del alcoholismo y en el proceso salud-enfermedad-atención-cuidado. Desde este enfoque, de mayo de 2020 a enero de 2021, se realizó un estudio cualitativo, en el que se entrevistó a diez mujeres elegidas bajo ciertos criterios y se realizó observación no participante en un grupo femenino Alcohólicos Anónimos. Entre los principales resultados, se reconoce una trayectoria de abuso de alcohol y su atención, concatenada con la trayectoria de cuidados. Este hallazgo delimitó la categoría de “ruptura en el cuidado” para develar el maltrato, la precarización de vida y salud de las mujeres y sus hijos e hijas.

## Abstract (en)

This article aims to analyze the experiences related to motherhood and care among mothers who attend mutual support groups to address alcohol-related harm in Mexico City and the State of Mexico. Drawing on the conceptual framework of collective health from a gender perspective, we contend that socioeconomic and gender-related factors influence the social determination of alcoholism and the health-disease-attention-care process. A qualitative study was conducted between May 2020 and January 2021, which included interviews with ten women who were selected based on specific criteria, as well as non-participant observation in a women’s Alcoholics Anonymous group. The main findings show how trajectories of alcohol abuse and its management are interconnected with trajectories of care. From there, it was possible to identify a “break in care,” a category that sheds light on mistreatment and the precariousness of life and health of women and their children.

## INTRODUCCIÓN

A nivel mundial, se registran 46 millones de mujeres con algún trastorno por el consumo de bebidas alcohólicas^1^. En México, los datos de 2015 indicaron que el consumo de esta sustancia en población general fue el principal factor de riesgo para desarrollar enfermedades como cirrosis, accidentes de tránsito, desórdenes mentales y consumo de otras sustancias y, en el caso de las mujeres, se indicó una mayor carga de enfermedades mentales, como los trastornos depresivos^2^. Desafortunadamente, el consumo de bebidas con alcohol también desencadenó sucesos fatales: en 2017, murieron 14.176 personas por enfermedad alcohólica del hígado^3^ y, en población femenina, la mortalidad por enfermedades del hígado que pudieran estar asociadas al consumo de alcohol fue la séptima causa de defunción en 2022^4^.

Por otra parte, y desde una perspectiva de género en salud, se ha develado el rol tradicional del cuidado que las mujeres deben brindar ante el abuso de alcohol de sus parejas varones o familiares^5^^,^^6^. También se han documentado las diferencias que por condición de género se constituyen en torno al consumo problemático. Por ejemplo, en los hombres, abusar de bebidas alcohólicas se considera como una conducta tanto esperada como tolerada en ellos, no siendo así en las mujeres, ya que si ellas beben en grandes cantidades se enfrentan con una doble estigmatización por ser mujeres e ingerir alcohol^7^^,^^8^^,^^9^^,^^10^^,^^11^. Estos hallazgos develan la expectativa social que les exige un menor consumo o la abstinencia a esta sustancia psicoactiva^12^.

Entre los daños asociados al alcohol que afectan a las mujeres, se identifica la violencia perpetrada contra ellas. En México, la intoxicación con bebidas alcohólicas por parte de las mujeres suele tomarse como una justificación para ofenderlas e incluso agredirlas^11^^,^^13^^,^^14^, especialmente si incumplen con las responsabilidades domésticas o parentales encomendadas^15^. A esto se añade la violencia de género que ejercen sus parejas con problemas por el alcohol. Según datos secundarios de la Encuesta Nacional de Adicciones 2011^16^, la violencia perpetrada por la pareja se presentó cuando uno de los integrantes, principalmente varones, ingirió esta sustancia, pero si ambos consumían, la posibilidad de tener un episodio violento en la pareja fue mayor.

En este marco, este artículo se centra en el reconocimiento de los daños en mujeres que consumen alcohol y son madres, ya que además de ser un tema poco abordado, se considera fundamental denotar las afectaciones que enfrentan, entre ellas, los señalamientos que las estigmatizan como madres negligentes o malas madres por consumir bebidas alcohólicas y, si su consumo ocurre durante el embarazo, el señalamiento es mayor, sufriendo un triple estigma^17^. Se considera que los mandatos tradicionales de género, como los expresados en el contexto mexicano, además de imponer en las mujeres la maternidad como un acto deseado, les asigna solo a ellas la responsabilidad de cumplir con tareas de crianza y cuidado de las hijas y los hijos desde el valor del “buen” ejemplo^18^^,^^19^, a pesar de vivir bajo determinadas situaciones que las vulneran, como el abuso del alcohol.

Por tanto, la condición de salud y los estereotipos de género inciden para que mujeres que son madres oculten los padecimientos, así como los daños asociados al alcohol, siendo esta una de las razones por las cuales, se retrasa la detección de su problema de salud o, en el peor de los casos, se les niega la atención o ayuda.

### La atención de mujeres madres frente al consumo de alcohol

Otra de las afectaciones que viven las mujeres madres al tener un consumo problemático de alcohol es la concerniente a la atención recibida, si bien se reconoce tanto la escasez de programas de tratamiento especializado con perspectiva de género, como las barreras que enfrentan para acceder a ellos^20^^,^^21^^,^^22^^,^^23^, también se identifica que son pocos los lugares donde las mujeres pueden expresar de forma segura las situaciones que las dañan a causa del consumo de esta sustancia. Dentro de las pocas alternativas disponibles para su atención se encuentran los grupos de apoyo mutuo^24^, dichas agrupaciones son un referente para la autogestión de la salud mental, ya que en estos lugares se visibilizan, desde las voces de las mujeres, las condiciones que las oprimen, maltratan y/o marginan^25^^,^^26^.

Para Menéndez^27^ los grupos de apoyo mutuo son una forma de autoatención y una alternativa organizada por los colectivos para dar respuesta o para enfrentar los problemas de salud que los afectan. De acuerdo con Menéndez^27^ y Ettorre^28^ es posible identificar dos modos de atención para los problemas por el consumo de alcohol: el primer modelo es el de tipo sanitario, fundamentado desde el enfoque médico hegemónico y, el segundo, es la autoatención. Además, las alternativas de atención organizadas se encuentran social e históricamente determinadas por la condición de género, la clase social, el grupo de edad, entre otros determinantes estructurales^29^.

Los antecedentes en México sobre el apoyo mutuo para los problemas por el consumo de alcohol hacen referencia a los grupos de 24 horas de Alcohólicos Anónimos. Una de las primeras agrupaciones en el país surgió en 1946 y, de acuerdo con Menéndez^30^ y Rosovsky^31^, su conformación ocurrió como una alternativa de atención ante la falta de programas de tratamiento para el alcoholismo de esa época pero, además, son una opción ante las limitaciones del abordaje biomédico en el tratamiento de los problemas por el consumo de alcohol^27^^,^^28^.

Los grupos tradicionales de Alcohólicos Anónimos se caracterizan porque en ellos se congregan, mediante juntas de hora y media, las personas afectadas por los problemas del alcohol. La organización entre integrantes de Alcohólicos Anónimos se basa en un principio de horizontalidad, en el que hombres y mujeres comparten las historias de sufrimiento asociadas al alcohol, así como también comunican su experiencia de recuperación. Los grupos de apoyo mutuo, entre otras organizaciones para la autoatención del consumo de sustancias en México, suelen retomar las guías de Alcohólicos Anónimos, en las que se incluye el programa de recuperación personal de 12 pasos. Como primera actividad, las personas que quieren dejar de beber, hacen un autorreconocimiento de la falta de control sobre el consumo de alcohol, así como de sus consecuencias; posteriormente, van practicando las demás etapas del programa hasta lograr compartir el mensaje de su recuperación^32^. 

Las 12 tradiciones, que también forman parte de la bibliografía, brindan orientaciones para mantener la unidad y organización de la comunidad de Alcohólicos Anónimos. En general, el programa ayuda a alcanzar y mantener la sobriedad de hombres y mujeres que asisten a los grupos^30^^,^^31^^,^^32^^,^^33^. Aunque, como se mencionó, la abstinencia al alcohol en las mujeres suele ser una expectativa social a cumplir, que limita la posibilidad de elegir un menor consumo, ya que se las considera incapaces de moderar el uso de bebidas alcohólicas^12^.

Por otra parte, si bien la investigación sobre las prácticas de recuperación del consumo de alcohol en los grupos de apoyo mutuo de Alcohólicos Anónimos han aumentado en México^30^^,^^31^^,^^32^^,^^33^, los estudios se han enfocado principalmente en varones, dado que se estima beben más y, por tanto, se argumenta que acuden más a este tipo de ayuda. Documentar la autoatención para los problemas por el consumo de alcohol desde la experiencia de los hombres -es decir, desde una mirada androcentrista- marca importantes sesgos de género^34^ en el estudio sobre los daños asociados al consumo de mujeres, así como de sus prácticas de recuperación, invisibilizando las experiencias y las desigualdades de género que las afectan. Al respecto, Scott^35^ sugiere que desestimar las experiencias de grupos históricamente excluidos, como los de las mujeres, mantiene sin cuestionamiento las condiciones sociales que los organizan de forma asimétrica y segregada. La propuesta teórica de la autora es reconocer y analizar la experiencia individual y colectiva, en este caso de las mujeres madres, como un medio para develar la condición histórica, social y cultural que sostiene determinadas formas de organización social.

Por esta razón, el presente trabajo pretende abonar al estudio sobre los problemas por el abuso de alcohol y la atención en población femenina, teniendo como principal objeto de interés evidenciar los daños que las mujeres que son madres viven frente al abuso de esta sustancia. Como refiere Gómez-Dantés^2^, se requiere fortalecer la investigación sobre la determinación social asociada a los problemas por el consumo de alcohol y su atención, particularmente, en grupos de mayor vulnerabilidad. 

Este artículo deriva de la investigación doctoral “Desigualdades en la respuesta social organizada para la atención de mujeres con daños asociados al consumo de alcohol”^36^. El estudio se ancló en el enfoque de la salud colectiva, la respuesta social organizada y la perspectiva de género^37^^,^^38^^,^^39^. Desde estos referentes teóricos se analiza la experiencia de las mujeres por ser ellas quienes, a través de sus prácticas de recuperación dentro de agrupaciones de apoyo mutuo como Alcohólicos Anónimos de México, han colectivizado sus experiencias de maternidad, las tareas de cuidado y su recuperación, entre otros relatos sobre los daños vinculados al abuso de alcohol. 

Derivado de todo lo anterior el objetivo del presente artículo es analizar las experiencias sobre la maternidad y los cuidados de mujeres madres que asisten a grupos de apoyo mutuo en la Ciudad de México y el Estado de México para autoatender los daños asociados al alcohol. 

## METODOLOGÍA

Se trató de un estudio cualitativo^40^, realizado en dos etapas, la primera fase fue de aproximación-exploración y la segunda de inmersión-profundización.

Teniendo como antecedente que las mujeres se enfrentan a mayores desventajas para recibir tratamiento especializado en el sector público de salud u otros modelos de ayuda, y con el propósito de asegurar la recolección de la información, se optó por contactar a mujeres madres que asistieran a grupos de apoyo mutuo de Alcohólicos Anónimos de 24 horas, ubicados en la zona oriente de la Ciudad de México y el Estado de México. 

Se realizó un muestreo intencional^41^^,^^42^ de diez mujeres, que asistían a grupos de 24 horas de Alcohólicos Anónimos para autoatender los problemas asociados al abuso de alcohol. Cuatro de ellas, que asistían a grupos mixtos de Alcohólicos Anónimos -es decir, formaban parte de agrupaciones integradas por hombres y mujeres- fueron entrevistadas vía telefónica durante la primera fase del estudio. En la segunda fase de la investigación participaron las otras seis mujeres, que fueron entrevistadas de forma directa en un grupo femenino de Alcohólicos Anónimos. Todas las mujeres cumplieron con los siguientes criterios de selección: ser madres, tener 20 años o más y haber transitado por el cuarto y quinto paso del programa de recuperación personal de Alcohólicos Anónimos. Se asumió que, al trascurrir por estos pasos del programa, las participantes habían reconocido el consumo de alcohol y los daños asociados. 

Se elaboraron dos guías de entrevista, una para cada fase del estudio. La información obtenida fue audiograbada, cada entrevista tuvo una duración de 60 a 120 minutos y contó con la firma del consentimiento informado. Los temas explorados fueron: las motivaciones que como mujer tuvo para consumir alcohol, los daños asociados, el alcoholismo como enfermedad, los mandatos de género para las mujeres como la maternidad, el trabajo en casa o de cuidados y el vínculo que estos tienen con sus experiencias de consumo o sobriedad, los momentos para pedir ayuda, las razones para acudir a la agrupación de Alcohólicos Anónimos, su proceso de recuperación y su recorrido por la atención sanitaria u otras formas de atender sus padecimientos. 

### Trabajo de campo

El trabajo de campo se realizó de mayo de 2020 a enero de 2021 y, dado el contexto de la pandemia por covid-19, se realizó en dos etapas. Como se mencionó, en la primera etapa las entrevistas se realizaron vía telefónica, ya que en ese momento la Jornada Nacional de Sana Distancia emitida por la Secretaría de Salud de México recomendó el cierre de lugares no esenciales, así como el confinamiento en casa. El primer contacto con las participantes se realizó con una mujer que asistía a Alcohólicos Anónimos y a quien la investigadora ya conocía. Una vez que se la invitó a participar en el estudio, ella facilitó la comunicación con las otras tres mujeres de grupos mixtos de Alcohólicos Anónimos para ser entrevistadas. 

En la segunda etapa del trabajo de campo, una vez que las autoridades sanitarias aprobaron el retorno paulatino a las actividades, se visitó a un grupo femenino de Alcohólicos Anónimos para invitar a las mujeres que allí estaban a participar en la investigación. Una vez que ellas aceptaron, se programó una fecha y se realizaron entrevistas individuales dentro de su agrupación. En esta fase participaron seis mujeres. Con el propósito de enriquecer la recolección de información, la investigadora asistió, previa autorización de las participantes, a cinco juntas de hora y media de la agrupación. Se empleó la observación no participante bajo una orientación etnográfica, así como conversaciones informales. El objetivo de las visitas fue conocer las experiencias colectivas que las mujeres compartían durante las juntas, así como identificar las prácticas-rituales de recuperación del alcohol. 

La Tabla 1 detalla las etapas del estudio, el número de participantes y las estrategias de recolección de información utilizadas.

**Tabla 1:** Etapas del trabajo de campo. Ciudad de México y Estado de México, 2020-2021.

Nombre de las etapas	Fecha	Número de participantes	Técnicas e instrumentos
Fase de aproximación-exploración	Mayo 2020	Cuatro mujeres entrevistadas telefónicamente, que asisten a grupos mixtos de Alcohólicos Anónimos	Guía de entrevista semiestructurada Diario de campo
Fase de inmersión-profundización	Septiembre 2020 a enero 2021	Seis mujeres entrevistadas presencialmente, que asisten a un grupo femenino de Alcohólicos Anónimos	Guía de entrevista semiestructurada Cinco visitas al grupo femenino de Alcohólicos Anónimos Observación directa no participante bajo una orientación etnográfica Conversaciones informales con las participantes Diario de campo

Fuente: Elaboración propia.

### Procesamiento y análisis de la información

A partir de un análisis artesanal de la información cualitativa^43^, como primer paso, se transcribieron las entrevistas de ambas fases del estudio. Los documentos finales fueron revisados en repetidas ocasiones con el propósito de clasificar y comparar la información obtenida. A partir de la constante comparación se elaboró una matriz temática en un archivo de Excel. Además de la codificación por temas, la información recolectada se organizó en calendarios de vida con la finalidad de visibilizar la temporalidad de determinadas experiencias vitales de las mujeres del estudio.

**Figura 1:**
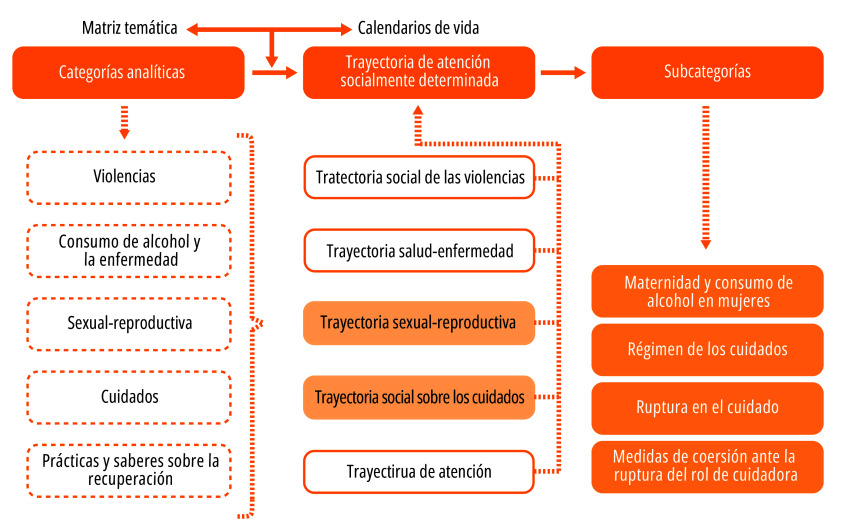
Estrategia para el análisis de la información, según categorías y subcategorías de análisis.

De este análisis surgieron las dos categorías-trayectorias centrales (Figura 1): la *trayectoria sexual-reproductiva* y la *trayectoria social sobre los cuidados*. De estas, se derivaron las siguientes subcategorías: 1) *maternidad y consumo de alcohol*; 2) *régimen de los cuidados*; 3) *ruptura en el cuidado*; 4) *medidas de coerción ante la ruptura del rol de cuidadora*.

Fuente: Elaboración propia.

El análisis se enriqueció con la triangulación de información de las conversaciones informales, las observaciones bajo una orientación etnográfica y las anotaciones hechas en el diario de campo^43^.

### Consideraciones éticas

Previo a las entrevistas, todas las mujeres participantes fueron informadas acerca de los objetivos del estudio, el carácter libre y voluntario de su participación, la confidencialidad de la información proporcionada y el resguardo de sus identidades, y firmaron el consentimiento informado, en el que también se solicitó autorización para audiograbar las entrevistas y publicar los resultados de la investigación. Para los términos del consentimiento informado, se consideraron los lineamientos del Reglamento de la Ley General de Salud en Materia de Investigación para la Salud, específicamente, el título segundo “De los Aspectos Éticos de la Investigación en Seres Humanos”^45^. Además, el proyecto contó con la conformidad del Núcleo Básico del Programa del Doctorado, de acuerdo con los lineamientos de la Universidad Autónoma Metropolitana Xochimilco^46^. Para asegurar el anonimato de las participantes del estudio se utilizaron nombres ficticios. 

## RESULTADOS

El método analítico permitió reconocer que la experiencia individual de las mujeres se interrelaciona con diversos acontecimientos, contextos, relaciones y motivos de las acciones^44^, de lo cual se desprendieron ciertos hallazgos emergentes. Primero, se reconoce que la trayectoria de atención y recuperación del consumo de alcohol se encuentra socialmente determinada al estar interconectada con las trayectorias de vida como las violencias, el consumo de alcohol como trayectoria salud-enfermedad, la maternidad como parte de la trayectoria sexual-reproductiva, la trayectoria sobre los cuidados y la trayectoria de atención. La contrastación de los calendarios de vida de las participantes evidenció su experiencia colectiva sobre los daños que como mujeres viven frente al consumo de alcohol.

Con relación al perfil sociodemográfico, las diez mujeres madres entrevistadas estaban dentro del rango de edad de 36 a 74 años, tenían un nivel socioeconómico bajo, y un promedio de tres hijas o hijos. Respecto al estado civil e ingreso económico: cinco mujeres vivían en pareja, una de ellas afirmó estar pensionada, y las otras cuatro dependían económicamente de sus esposos, los cuales no contaban con un trabajo formal; las otras cuatro mujeres estaban separadas y sus ingresos provenían de trabajos eventuales e informales; la última mujer había enviudado y dependía económicamente de familiares cercanos. Con relación al nivel de estudios, dos mujeres habían estudiado la primaria, cuatro habían concluido la secundaria, tres habían cursado el nivel medio superior y una tenía una carrera profesional. Todas las mujeres consideraban que su principal ocupación era el trabajo en el hogar como “amas de casa”. 

Los resultados develaron que, en promedio, las mujeres de esta investigación consumieron alcohol durante al menos 16,8 años antes de buscar alguna forma de ayuda, mientras que el número de años en sobriedad dentro de los grupos de apoyo mutuo fue de 16,2, según lo reportado al momento de la entrevista. Estos datos mostraron que las mujeres tardaron bastantes años en solicitar o recibir algún tipo de apoyo y en sufrir los daños asociados al abuso del alcohol, teniendo distintas pérdidas de acuerdo con los hallazgos de las cuatro subcategorías analíticas. 

### Maternidad y consumo de alcohol en mujeres

Las experiencias sobre el consumo de alcohol en las mujeres de este estudio estuvieron vinculadas a eventos significativos, como el inicio de su vida sexual, la maternidad temprana, la lactancia y los cuidados. Todas las mujeres eran madres: cinco dijeron haber tenido su primera relación sexual, su primer embarazo y vida en pareja antes de los 20 años, por lo que vivieron un embarazo a temprana edad. En las mujeres del estudio, el embarazo precoz se vinculó con otros determinantes sociales, como los señalados por la Secretaría General del Consejo Nacional de Población^47^: baja escolaridad, inicio temprano de la vida sexual sin protección, poco uso de métodos anticonceptivos en la primera relación sexual, matrimonio infantil y falta de un proyecto de vida. Una de las mujeres así refirió este acontecimiento:

*“No me figuraba ni tener hijos, ni casarme, el papá de mi hija, pues los dos tomábamos, éramos como esos amigos de farra ¿no?, con los que te podías ir a la disco, ir, regresar, entonces este… en una de esas ocasiones empezamos a tener relaciones él y yo, sin protección, entonces este… pues fue pasando el tiempo y cuando ya no me baja como la regla, pues entonces es cuando yo me doy cuenta de que estoy embarazada”.* Esperanza, Ciudad de México

Además, los relatos permitieron identificar que, desde la percepción de las mujeres, el embarazo fue solo su responsabilidad y, si bien en algún momento tuvieron la expectativa de estar acompañadas por una pareja^48^^,^^49^, la experiencia de la maternidad, además de ser impuesta, terminó por vivirse con una fuerte carga emocional, de forma solitaria y como un hecho inevitable. Así lo explicó la siguiente mujer:

*“Yo andaba… en el cotorreo, en las fiestas y todo y tuve pues un noviecillo a esa edad, y pues en el cotorreo yo salí embarazada, lo cual yo no quería… al principio su papá de ella* [de la hija de la mujer entrevistada] *pues se hizo responsable y ya después… pues él siguió su cotorreo y se fue”.* Alegría, Estado de México.

Otra circunstancia de enorme presión para las mujeres fue la exigencia, por parte de sus familiares o personas cercanas a ellas, de mantenerse en abstinencia mientras cursaban el embarazo, la falta de control del consumo de alcohol a causa de su posible dependencia física a la sustancia, provocó que se las culpara y responsabilizara de los daños ocasionados a sus criaturas. Fue preocupante identificar que, durante la atención prenatal en el sector salud, el personal sanitario no brindó una orientación adecuada, más grave aún fue denotar que el personal médico, una vez que reconoció los problemas asociados al consumo de alcohol durante la etapa de embarazo, reforzó el discurso sancionador hacia la mujer que lo ingiere. Dicha exigencia develó que la maternidad, ya sea en el espacio familiar o en el ámbito sanitario, no necesariamente fue un proceso de acompañamiento o de orientación para el cuidado de la salud de las mujeres y sus hijas e hijos; por lo contrario, se identificó como un evento vigilado y controlado para alcanzar una forma esperada de ser mujer y ser buena madre independientemente de las desventajas vividas^49^^,^^50^. Así lo compartió una de las participantes:

*“Desde que salgo embarazada me atendía en el centro de salud, ahí este me atendía* […] *cuando iba yo a visita pues me aguantaba porque no bebía y realmente pues los doctores pues nunca se daban cuenta que yo iba ebria… nunca me dijeron respecto al ¡no, ya no beba!* […] *En el último embarazo pues sí me espantaron* […] *dentro del hospital* [en el Hospital de Perinatología] *me dijeron que… que mi bebé venía mal por causa del alcohol porque yo a veces pues tomaba el alcohol y a veces no tenía para tomar alcohol y tomaba agua y así y que esas complicaciones se le iban pues al bebé… por eso, es por lo que el bebé iba a nacer con hidrocefalia, me decían”.* Alegría, Estado de México

En los relatos anteriores es posible ver cómo se va reproduciendo el acuerdo social del mandato que asigna a las mujeres la responsabilidad de asegurar el estado de salud de sus hijas e hijos, y muestra los obstáculos para acceder a la más mínima orientación médica o apoyo de algún otro familiar cercano ante el consumo de alcohol durante el embarazo. La ideología que las define como personas dotadas de forma biológica y hormonal para procrear, parir, amamantar y cuidar^51^ profundiza las desventajas para atender sus problemas de salud, y naturaliza la responsabilidad de la crianza, culpando solo a ellas de lo que les pase a hijos e hijas. Esta concepción minimiza la participación de la pareja y, como se mencionó, desdibuja el contexto de desventaja y precariedad en el que han vivido estas mujeres. 

Con relación al periodo de lactancia, culturalmente las mujeres viven bajo presiones sociales para cumplir con el trabajo de amamantar y proveer de defensas al sistema inmunológico del recién nacido^50^, derivando en constantes cuestionamientos y descrédito para quienes no cumplen con su función natural^52^. De acuerdo con varias autoras, como Gutiérrez^52^, Lazarre^53^ y Calaffel^54^, el periodo de lactancia exige en las mujeres afrontar nuevos cambios en su cuerpo, así como la reorganización de su tiempo. En las mujeres con problemas por el consumo de alcohol, esta demanda nuevamente las coloca en el dilema de continuar bebiendo (ser malas madres) o amamantar (ser buenas madres). Las dificultades se suman cuando ellas tienen que continuar con el proyecto de la buena maternidad y la crianza, debiendo olvidar el espacio de convivencia y de fiesta, para limitarse a un espacio confinado, solitario, lleno de culpas y ansiedad. Una de las mujeres lo explicó así:

*“Cuando yo vuelvo a beber, decido ya dejarla de lactar… porque pues pensaba que, si yo tomaba y le daba de, de pecho ¿no? pues algo se le iba a poder trasmitir… Hubo una época en donde empiezo a sentir esa necesidad de querer controlar el alcohol, ya había algo como importante, que tenía que cuidar. Fue todo un desastre, porque ya no era como la chica que se iba a una fiesta y se emborrachaba, ahora era como la, la mamá y era como la esposa, pero yo empezaba a sentir mucha ansiedad y entonces esa ansiedad la empiezo a desahogar… bebiendo, pero ya en la casa… a solas, ya asegurándome que mi hija estuviera como, como segura… y empiezo a tomar más”.* Esperanza, Ciudad de México

Por último, de acuerdo con Stern^18^, el embarazo adolescente en contextos menos favorecidos suele ser visto como un camino para obtener prestigio social y respetabilidad. Otros estudios^55^^,^^56^^,^^57^ coinciden en que la maternidad en contextos de vulnerabilidad es vista como el camino para alcanzar un estatus social, quizá este acontecimiento llegó a ser visto por las mujeres del estudio como un proceso que les brindaría la oportunidad de borrar las desventajas sociales y de género en las que habían vivido; sin embargo, y como se evidenció en los relatos, las desventajas solo se profundizaron. 

### El régimen de los cuidados

Los cuidados son actividades y prácticas enmarcadas como trabajo orientado a la reproducción y sostenimiento de la vida. Para Bathyány^58^, estas labores permiten reparar la fuerza de trabajo de los grupos humanos; sin embargo, su asignación como actividad social se sostiene desde estructuras culturales y económicas que, en todo caso, reproducen la ideología de la distribución del trabajo a partir de las características sexuales de las personas. De forma concreta, asignan labores -productivas- específicas para hombres, así como funciones y actividades -de reproducción- particulares para las mujeres, configurando un régimen de cuidados, basado en la desigualdad^59^. 

Como se identificó, el embarazo, la maternidad, la lactancia y los cuidados, además de ser mandatos sociales, se asumieron como un ideal romantizado de lo femenino, de hecho, estas labores estaban profundamente arraigadas en las mujeres que, aún después de alcanzar la abstinencia, siguieron viendo estas tareas como su responsabilidad, demandándoles la reorganización de su tiempo para cumplir con la carga de cuidados, al mismo tiempo que trabajaban en la recuperación de su salud, tal como se enuncia en el siguiente relato:

*“Yo tengo un evento* [en el grupo de Alcohólicos Anónimos], *por ejemplo… ya sé que el domingo, porque ya está programado, ¡yo necesito estar a las diez de la mañana! Ok, desde el sábado compro la comida, o lavo en la noche o me levanto muy temprano, dejo mis actividades bien ¡ahora sí este tiempo es mío! Yo pienso que, como mujeres, la verdad somos como… muy responsables”.* Laura, Estado de México

Las experiencias sobre la asignación de cuidados en las mujeres develaron el régimen de cuidados reproducido. Bajo esta demanda social, se perpetúa la ideología heteropatriarcal con la cual se sostiene el reparto asimétrico de las tareas de cuidados materiales -como preparar la comida, la organización del aseo doméstico, entre otras labores- y los cuidados afectivos, como dar el buen ejemplo y/o brindar estabilidad emocional a hijas e hijos o pareja^60^^).^

### La ruptura en el cuidado

El concepto de ruptura ha sido empleado en esta investigación para hacer referencia a la trasgresión del ideal femenino sobre la maternidad y los cuidados que las mujeres deben cumplir^61^. Para Rosero^49^, el concepto de ruptura ayuda a discutir las formas hegemónicas de maternidad, permitiendo cuestionar los mandatos tradicionales de género, como los cuidados impuestos a mujeres que son madres, así como las condiciones socioestructurales sobre las cuales se les demanda este papel. Este subapartado tiene el propósito de destacar las contradicciones vividas por las participantes de esta investigación al momento de intentar cumplir con el régimen de cuidados, mientras enfrentaban de forma estigmatizada y solitaria los daños por el abuso del alcohol.

El trabajo de los cuidados en la esfera privada y familiar tiene dos características principales: la primera, no son remunerados y, la segunda, son actividades invisibilizadas. La primera característica se intenta justificar al asumir que los cuidados son una ocupación natural y cotidiana de las mujeres, razón por la cual no debe tener otra forma de reconocimiento social más que el pago con amor o gratitud por parte de quien recibe los cuidados. La condición de estas labores oculta y sobrevalora la contribución de las mujeres en la vida social; sin embargo, paradójicamente, cuando no se cumple con estas tareas, el cuidado se visibiliza porque se deja de cubrir una necesidad^62^. Entonces, las personas encargadas de realizarlos, en este caso las mujeres, quedan bajo los señalamientos de ser malas esposas, ser malas madres, imperfectas o negligentes, porque rompen con una norma social^49^. Por supuesto que esto no significa una desestimación de la integridad y seguridad de la persona que tiene el derecho a ser cuidada.

Bajo los argumentos anteriores, se abordó la categoría *ruptura en el cuidado*. Es importante precisar que en este trabajo se hace referencia al concepto de ruptura en dos sentidos. El primero, ubica los momentos en los que ocurrió la “trasgresión” de este mandato social, es decir, cuándo se quebrantó el régimen de los cuidados poniendo en jaque el papel como mujeres madres, pues las propias participantes consideraron que eran ellas quienes “amenazaron” el bienestar de sus hijos e hijas. En un segundo sentido, se empleó el concepto de ruptura para dar nombre a los acontecimientos que pusieron en mayor riesgo y vulnerabilidad tanto a las mujeres como a sus hijos e hijas durante los momentos más críticos, a causa del abuso del alcohol.

La propuesta de nombrar la ruptura en el cuidado también sirvió para develar las etiquetas sociales con las cuales se define la identidad de las mujeres que han vivido estas experiencias, fórmulas utilizadas para vigilar, controlar, juzgar, enjuiciar, discriminar y marginar a las mujeres que deben cumplir con formas hegemónicas de maternidad y cuidado mientras sufren los daños por el alcohol, ocultando las desventajas sociales y materiales en la determinación del abuso del alcohol, exigiéndoles, contradictoriamente, el cumplimiento de estos mandatos, viviendo en condiciones de pobreza, desventaja social, opresión, más sus problemas de salud y las brechas en su atención.

Bajo la propuesta conceptual de la ruptura en el cuidado, fue posible escuchar y comprender las experiencias silenciadas por las mujeres a causa de la vergüenza que les generó verse incapacitadas por sus problemas de salud para cuidar de ellas mismas o cuidar de sus criaturas. Los resultados en este estudio de caso muestran que las mujeres no lograron recibir ninguna forma de ayuda y que la falta de respuesta familiar -como primer grupo de apoyo- o el desconocimiento de los familiares sobre el consumo de alcohol -como un problema de salud- provocaron que los daños no solo fueran mayores, sino irreversibles. La muerte de niñas y niños, como afectación permanente, sumado a las dificultades para brindar o recibir apoyo y cuidado familiar, social o institucional fueron varias de las experiencias vividas. Una de las entrevistadas compartió su experiencia al conocer a otras mujeres que sufren estas rupturas.

*“Había una compañera que me compartía cómo había perdido a su hijo, y que su mayor fondo de sufrimiento o sea había sido el hecho, de ir como a su tumba y llorarle y entonces yo decía ¡no quiero vivir como esas situaciones!, porque pues su hijo murió como, como por producto de, pues de una borrachera, o sea tuvo un accidente el niño y cuando ella despertó o sea el niño ya no tenía vida y entonces ese tipo como de experiencias, eran las que yo decía ¡no! Por dentro sabía que… que a lo mejor sí iba a llegar a algo así, porque yo perdía la conciencia”.* Esperanza, Ciudad de México

Como respuesta a la ruptura de los cuidados, las mujeres son objeto de agresiones verbales y/o físicas y reclamos. Estas respuestas invisibilizaron los riesgos a los que ellas y sus hijos e hijas estuvieron expuestos y obligó a las mujeres a afrontar en solitario los cuidados y la dificultad para controlar el consumo del alcohol. Así lo narró una de las mujeres:

*“Yo tenía mucho la costumbre de bañarlos por la noche* [a los hijos], *entonces como yo todo el día realmente me la pasaba tomando, en una ocasión a una niña la bañé en la noche y yo como ya andaba bien bebida… pues yo me acosté en la cama y me quedé dormida y la dejé sentadita en el agua, hasta que llegó mi esposo y que me levanta de las greñas y todo, yo ahí comprendo que… pues fue su reacción de él del coraje de ver cómo estaba la niña entonces… pues ya también a su mamá le dijo: ¿qué no te das cuenta que la deja?, ¡pues es que ella se encerró!* [contestó la mujer] *Y ahí hasta que llegó él pues ya la vistieron y todo yo bien, bien tomada estaba ahí… Y también… una vez le cambié el pañal y por entretenerla o sea mi inconciencia de que andaba yo tomada, le cambié el pañal, le estaba cambiando el pañal y le doy unas monedas en la mano* […] *y se le va una moneda de cincuenta centavos a su boquita, entonces la niña ya se estaba ahogando y yo no hallaba ni qué hacer, entonces mi esposo la agarró y le empezó a pegar en la espalda y le metió el dedo y le sacó la moneda y ya hasta le estaba saliendo sangre de la nariz, mi hija y le dije yo después y con eso dije: ¡ya no voy a tomar, ya no voy a beber, ya no voy a beber! pero… otra vez… vuelvo a empezar otra vez…* Alegría, Estado de México

### Medidas de coerción ante la ruptura del rol de cuidadora

Los cuidados generan tensiones entre quienes deben otorgarlos, quienes deben recibirlos y quienes van vigilando su cumplimiento. Dichas tensiones guardan sus propios riesgos^49^ como son la coacción, el maltrato y el abuso. En el caso de las mujeres que no cumplieron con su rol como cuidadoras, la coacción se concretó en su señalamiento, en amenazas y, en algunos casos, se las expulsó del hogar o simplemente se decidió que otra mujer quedara al cuidado de las y los menores. La separación de sus hijas e hijos se demostró como una sanción social digna de merecer por no saberlos cuidar. Varias mujeres así lo vivieron:

*Al principio pues me sentía yo mal, porque decía ¿cómo es posible de que… de que no me quieran entregar a mi hijo…? Me lo quitaron porque por lo mismo de cómo me veían, entonces este… ahí influyó mi suegra también, me dijo: ¡no pues mejor que se quede porque si no cómo le va a hacer con su tomadera y él¡* Alegría, Estado de México

Como otra medida de coacción también se las condicionó al encierro en algún anexo, lugares que suelen operar fuera de la normatividad indicada por la Secretaría de Salud. Son centros que brindan ayuda a personas con problemas por el consumo del alcohol u otras sustancias. Sus métodos de recuperación tienen como principal característica el aislamiento o encierro de las personas “adictas” durante un periodo de treinta a noventa días^63^. Las experiencias de algunas de las mujeres evidenciaron que ellas vivieron este tipo de sanciones y que su aislamiento las despojó de su rol^64^ como madres, intercambiando, bajo la presión de otros, su identidad por el de alcohólicas en la idea de la incompetencia para cumplir con las tareas de cuidado. Una de las participantes contó la forma en que fue coaccionada para aceptar esta forma de ayuda:

*¡La única manera en que te podemos ayudar es que tú te internes! si tú no quieres acceder a esto… pues a tratarte, me decían que se iba a iniciar un proceso para quitarme a mi hija, un proceso legal.* Esperanza, Ciudad de México

La siguiente mujer así narró su situación:

*Los únicos que me iban a ver* [al anexo] *eran mi suegra y mi marido y uno de mis cuñados por parte de mi hermana… fueron los que me iban a ver, mis hijos nunca me fueron a ver porque ellos mismos no querían… bueno mi suegra no quería, ni mi marido no querían que fueran a ver dónde estaba.* Alegría, Estado de México

En el interés por proteger a las y los menores intervino el cuidado de las abuelas o mujeres hijas. Este hecho, si bien demuestra relaciones de solidaridad que pueden surgir entre ellas, finalmente perpetúa el mandato del cuidado en las mujeres -y la desigualdad entre los géneros- para eximir a los hombres de la responsabilidad de proteger a otras personas, especialmente las más vulneradas. Así lo compartieron dos de las entrevistadas:

*“Les daba de comer* [a hijos e hijas] *y así, luego a veces estaba toda tomada y les daba de comer* […] *si ya… de plano ya estaba muy tomada, mi suegra era la que les daba de comer.* Alegría, Estado de México

*“Mis hijas, sobre todo la mayor, que fue a la que más daño le causé, porque ella se quedó ocupando el papel de madre, pues ella cuidaba a mis otros dos hijos”.* Lorena, Estado de México

Como consideración final, la razón de explicar la ruptura en los cuidados no solo surgió de la información compartida por las mujeres, sino por el interés de visibilizar las sanciones y riesgos a las que quedaron expuestas junto con sus hijos e hijas menores. Como se señaló en los relatos, la falta de cuidado por parte de ellas estuvo severamente sancionada por la familia y la comunidad en su conjunto, esta situación agravó su condición de desigualdad e incidió en el deterioro de su salud y bienestar. 

Todas las mujeres coincidieron en afirmar que su necesidad de pedir o recibir la ayuda surgió una vez reconocida la *ruptura en los cuidados*. Si bien estos hechos fueron un punto decisivo para enmarcar su trayectoria social de atención, este proceso se inició bajo la incertidumbre, la culpa y el desconocimiento para controlar o detener el consumo, más grave aún fue no poder identificar los lugares o instituciones en los que podrían ser ayudadas. Una mujer así lo relató:

*“Mis hermanos me decían que ya no bebiera tanto, que era una borracha* […] *pues ellos* [los hermanos de la mujer entrevistada] *me querían ayudar y decirme que no bebiera, pero no me decían cómo* […] *Llegó un momento que ya no quería beber, como le dije hace un rato, por mis hijos, porque ellos ya iban creciendo más y me decían que pues que no bebiera y yo también ya no quería, yo ya no quería beber, ya no… porque realmente a mí, ya me hacía mucho daño y dije… ¡no!, ¡es que yo ya no quiero beber, pero no puedo, no sé cómo!, ¿cómo le voy a hacer?* María, Ciudad de México

En este último testimonio se puede advertir la carencia de opciones o de información sobre los modos de atención que brindan tratamiento o ayuda a las mujeres madres que han vivido los daños por el consumo de alcohol. Además, resulta complicado identificar quién las puede apoyar.

Como se identificó, las respuestas de cuidado generalmente están concebidas como tarea femenina a realizarse en el ámbito privado, por ejemplo, cuando la mujer madre se enferma, el cuidado de sus hijos e hijas menores queda bajo la responsabilidad del grupo familiar; sin embargo y, como se mostró, en el caso de las mujeres madres que abusan del alcohol las primeras respuestas estuvieron pensadas bajo la lógica de la coerción, el maltrato o el abandono^65^^,^^66^. A esto se sumó la carencia de respuesta institucional. 

## DISCUSIÓN

Entre los diversos aspectos a discutir, el primero se relaciona con las perspectivas teóricas empleadas. El enfoque de género en el estudio sobre los problemas por el consumo de sustancias psicoactivas indica, en concordancia con los hallazgos de este artículo, que la condición de género en las mujeres incide en la forma en cómo se viven los problemas por el alcohol y el apoyo o atención que reciben, el proceso salud-enfermedad-atención-cuidado desde la mirada de la salud colectiva. 

Respecto al enfoque de género, se evidenció que las afectaciones por el consumo de alcohol en las mujeres se han subestimado^5^. Algunos de los estudios realizados en México, han evidenciado los daños en diferentes contextos de desigualdad. Por ejemplo, Natera y Casco^5^ mostraron la responsabilidad asignada a las mujeres para cuidar de familiares con problemas por el alcohol; Rodríguez^14^ por su parte, indicó que las violencias hacia mujeres trabajadoras sexuales se justifican por el trabajo desempeñado y por el consumo de alcohol, aunque sea esta sustancia un medio empleado por ellas para sobrellevar su condición de vida. 

En el caso de la atención que reciben, Romero^22^ documentó los obstáculos para el acceso a tratamientos especializados en adicciones para mujeres en reclusión, en otros estudios Lozano-Verduzco, Romero-Mendoza, Marín-Navarrete^67^ y Galaviz^68^ develaron la violencia de la que son objeto las mujeres al intentar su recuperación en centros que operan fuera de la normatividad. Además, Galaviz^68^ indicó que los programas de rehabilitación tienden a reproducir roles tradicionales de género, en los que el cumplimiento de parámetros femeninos -como el ser buenas mujeres- se toman como una señal de recuperación del consumo de sustancias psicoactivas, bajo estos mandatos se perpetúan las inequidades de género.

Por otra parte, el abordaje de la salud colectiva permitió identificar que la condición de género y socioeconómica actúan en la determinación social del proceso salud-enfermedad-atención-cuidado que reciben las mujeres madres ante su abuso de alcohol, y como hallazgo emergente se obtuvo el análisis crítico sobre el régimen de los cuidados. Otro punto a destacar es que aún quedan líneas de investigación pendientes por explorar, ya que al admitir que las mujeres son una categoría de análisis heterogénea^69^ se requiere estudiar los daños por el consumo de alcohol en mujeres que viven bajo determinadas condiciones de vida, como lo es la edad, la condición socioeconómica, el lugar de residencia, su pertenencia étnica, el tipo de trabajo que desempeñan, entre otros determinantes sociales del proceso de salud-enfermedad-atención-cuidado. En este sentido, se reconoce que los problemas por el abuso de alcohol en las mujeres son un problema de salud de mayor complejidad, que ocasiona afectaciones graves e incluso daños irreversibles hacia ellas. 

El siguiente punto de discusión tiene que ver con los hallazgos de este trabajo. Los resultados evidenciaron la falta de respuesta o escaso apoyo institucional y familiar hacia las mujeres madres que enfrentan problemas por el consumo de alcohol. Esta información confirma el papel de cuidado que se espera que las mujeres brinden a sus familiares^5^, siendo la razón por la cual ellas no reciben el apoyo oportuno cuando ingieren esta sustancia de forma problemática, a lo que se suma la violencia sistemática perpetrada contra ellas^13^^,^^16^. 

Desafortunadamente, las afectaciones hacia las personas que dependen de los cuidados de mujeres con problemas por el abuso de alcohol, y de los cuidados que ellas mismas requieren, son un tema poco estudiado, y esto mismo sucede con la investigación sobre la atención que reciben. Como se indicó, existen sesgos de género, que subestiman las experiencias de las mujeres al considerarlas como un grupo minoritario o menos afectado^35^, excluyéndolas también del estudio sobre las prácticas de recuperación del alcohol desde la autoatención. 

Entre los hallazgos, también se identificó que las afectaciones que las mujeres vivieron estuvieron interconectadas con diversos contextos de desigualdad social y de género, tales como la maternidad forzada en una edad temprana, los cuidados de los hijos en espacios solitarios y culpabilizados, la condición socioeconómica baja, los pocos años de instrucción escolar, más una serie de desventajas como la estigmatización, la opresión y violencias por no cumplir con parámetros sobre la “buena” maternidad -resultado concordante con Galaviz^68^- así como las expresiones de violencia por ser mujeres con problemas por el abuso del alcohol que incumplen con las tareas de cuidado. 

El último punto de discusión se relaciona con el número de años que las personas tardan para recibir alguna forma de atención. De acuerdo con Natera et al.^70^, en México suelen pasar hasta doce años para que las personas con consumo problemático de alcohol busquen ayuda. Sin embargo, y como se mostró en este estudio, las mujeres madres ingirieron bebidas alcohólicas durante al menos 16 años antes de buscar o recibir alguna forma de apoyo. Esta situación, entre otros determinantes sociales, seguramente profundizaron los daños en las mujeres madres y en quienes dependían de sus cuidados. 

## CONCLUSIONES

El cuidado es históricamente concebido como una tarea femenina a realizarse dentro del espacio privado y sin colectividad. Al no cuestionar esta idea, se asume una forma hegemónica de ser madre, de tal manera que si no se cumple con este mandato social las mujeres pueden verse señaladas. Cuando las mujeres tienen problemas por el abuso de alcohol suele sometérseles a sanciones, encierro, o simplemente son estigmatizadas y discriminadas por ser “malas madres”, en casos más graves se justifican las violencias ejercidas contra ellas. 

Se concluye que, al asumir la mirada hegemónica de la buena maternidad y los cuidados como actividades exclusivas de las mujeres, se invisibilizan las desigualdades de género y materiales, así como la falta de respuesta oportuna por parte del Estado y/o la carencia de redes de apoyo sociofamiliar ante la atención que requiere una mujer al enfrentar los daños asociados al abuso de alcohol. Dicha condición precariza su condición de vida y las de sus hijos e hijas menores, perpetuando la injusticia, la falta de oportunidades y la falta de acceso para recibir ayuda o servicios sanitarios para el cuidado y la recuperación de su salud, dejándolas socialmente excluidas y expuestas para vivir con mayores “daños, la violencia y la muerte”^71^^).^

Por otra parte, cabe decir que la investigación tuvo un diseño cualitativo que, si bien no pretende generalizar los hallazgos, los resultados permiten pensar en aquellas otras mujeres que al igual que las participantes de esta investigación, viven bajo condiciones similares y que posiblemente estén en situaciones de vulnerabilidad como las reportadas en el presente estudio de caso. 

Los resultados obtenidos proponen la categoría analítica *ruptura en el cuidado*, pero también deben ser vistos como un punto de apertura para destacar que las mujeres madres con daños por el abuso de alcohol requieren de una atención prioritaria y particular, al igual que las personas que se encuentran cercanas a ellas. Bajo esta línea argumentativa, los programas institucionales requerirían ser diseñados desde una perspectiva de género, con enfoque en los derechos humanos, en los que se reconozca el derecho de que sean cuidadas tanto ellas como los hijos y las hijas menores o personas que dependan de ellas, por lo tanto, se deberían erradicar las barreras de acceso a tratamientos y proporcionar información sobre los programas que contemplan la abstinencia al alcohol o, en todo caso, optar por un programa de reducción de riesgos y daños^12^.

Por último, hay que fortalecer la investigación desde un enfoque en el que la atención sea vista como parte del proceso salud-enfermedad-atención-cuidado, concibiendo la atención como una respuesta organizada y colectiva que permita enfrentar los padecimientos de salud. También es necesario considerar el enfoque de género, siendo este el argumento por el cual la investigación requiere ampliarse más allá del campo de los servicios sanitarios que, dicho sea de paso, son modelos de atención en los que se ha documentado que las mujeres tienen un menor acceso. En razón de lo anterior, existe la necesidad de aportar al conocimiento desde el estudio de las prácticas y saberes de recuperación o autoatención^27^ que gestionan las mujeres madres que han vivido los daños por el abuso de alcohol, pues a partir de las experiencias narradas desde estos espacios de seguridad para ellas, es como la investigación nombró la categoría sobre la ruptura en el cuidado.
